# DECOMICS, a shiny application for unsupervised cell type deconvolution and biological interpretation of bulk omic data

**DOI:** 10.1093/bioadv/vbae136

**Published:** 2024-09-20

**Authors:** Slim Karkar, Ashwini Sharma, Carl Herrmann, Yuna Blum, Magali Richard

**Affiliations:** IBGC, UMR 5095, University of Bordeaux, CNRS, Bordeaux Bioinformatic Center, Bordeaux 33077, France; Health Data Science Unit, Medical Faculty Heidelberg and BioQuant, Heidelberg 69120, Germany; Health Data Science Unit, Medical Faculty Heidelberg and BioQuant, Heidelberg 69120, Germany; IGDR (Institut de Genetique et Developpement de Rennes), UMR 6290, ERL U1305, Equipe Labellisée Ligue Nationale contre le Cancer, University of Rennes, CNRS, INSERM, Rennes 35000, France; TIMC, UMR 5525, Université Grenoble Alpes, CNRS, Grenoble F-38700, France

## Abstract

**Summary:**

Unsupervised deconvolution algorithms are often used to estimate cell composition from bulk tissue samples. However, applying cell-type deconvolution and interpreting the results remain a challenge, even more without prior training in bioinformatics. Here, we propose a tool for estimating and identifying cell type composition from bulk transcriptomes or methylomes. DECOMICS is a shiny-web application dedicated to unsupervised deconvolution approaches of bulk omic data. It provides (i) a variety of existing algorithms to perform deconvolution on the gene expression or methylation-level matrix, (ii) an enrichment analysis module to aid biological interpretation of the deconvolved components, based on enrichment analysis, and (iii) some visualization tools. Input data can be downloaded in csv format and preprocessed in the web application (normalization, transformation, and feature selection). The results of the deconvolution, enrichment, and visualization processes can be downloaded.

**Availability and implementation:**

DECOMICS is an R-shiny web application that can be launched (i) directly from a local R session using the R package available here: https://gitlab.in2p3.fr/Magali.Richard/decomics (either by installing it locally or via a virtual machine and a Docker image that we provide); or (ii) in the Biosphere—IFB Clouds Federation for Life Science, a multi-cloud environment scalable for high-performance computing: https://biosphere.france-bioinformatique.fr/catalogue/appliance/193/.

## 1 Introduction

Identification of the cell composition contributing to bulk molecular signals is a major challenge in molecular analysis in various applications, including cancer research (Nguyen *et al.* 2024). The development of in silico deconvolution methods has made it possible to revisit existing bulk omic data from large patient cohorts with regard to intra-sample heterogeneity, and thus to compare sample cell composition with available clinical annotations such as treatment response. Both supervised (Avila Cobos *et al.* 2020) and unsupervised (Onuchic *et al.* 2016, Sompairac *et al.* 2019, Chen *et al.* 2020, Qin *et al.* 2020, Kang *et al.* 2021) deconvolution methods have been proposed in the literature. Supervised methods estimate the component proportions using known cell-type reference matrices, whereas unsupervised methods estimate both the reference profiles and the component proportions, without prior knowledge except for the number of components to be considered. Supervised approaches are therefore limited by the quality of the reference signatures, while unsupervised approaches present difficulties in interpreting the inferred components and estimating the number of components to be considered. An intriguing advantage of unsupervised methods is that, unlike supervised methods, they can identify new cell populations or populations that would not have been taken into account a priori. Unsupervised deconvolution approaches have recently been used to identify radiogenomic signatures to predict prognosis of colorectal cancer (Zhong *et al.* 2022), to identify cellular compartments in unknown tumoral samples (Peng *et al.* 2019), or to infer clinical outcomes in melanoma patients (Nazarov *et al.* 2019). Applying unsupervised deconvolution approaches to patient cohorts is now possible thanks to recent advances in high-throughput sequencing that have generated an enormous amount of transcriptomic data as well as numerous methylome data. This is particularly true in the field of oncology, where more and more biological samples are being sequenced to help with patient stratification and prognosis. However, analyzing this type of data requires professional coding skills, which are rarely available to clinicians. In order to appeal large data analysis to a wider audience, user-friendly alternatives have been devised, including the development of the R-shiny package, which enables interactive web applications to be built using the R statistical and data mining software (Jia *et al.* 2022). If web applications exist to apply supervised algorithms on clinical datasets (Li *et al.* 2020), exploration of unsupervised algorithms has been so far limited to bioinformaticians with computing skills and does not provide guidance for biological interpretation of their outputs. Here we propose DECOMICS, a user-friendly shiny interactive web application designed to perform unsupervised deconvolution on transcriptomic and methylome (DNAm) data. Six different unsupervised methods are implemented in DECOMICS, including the most commonly used [independent component analysis (ICA) and non-negative matrix factorization (NMF)], and more recent algorithms [CDSeq; Kang *et al.* 2021), debCAM (Chen *et al.* 2020), PREDE (Qin *et al.* 2020), and EDec (Onuchic *et al.* 2016)]. Our tool also provides guidance during the process and helps with the biological interpretation of the results, which should be of significant interest for both bioinformaticians and clinicians.

## 2 Software description

**Figure 1.**
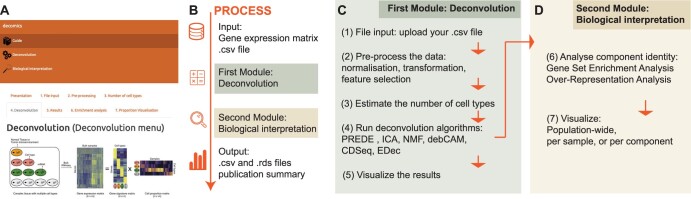
An overview of DECOMICS workflow to perform deconvolution of omics data. (A) Screenshot of the application. (B) Summary of the DECOMICS process. (C) Workflow of the module “Deconvolution.” (D) Workflow of the module “Biological interpretation.”

DECOMICS is a Shiny application available as an R package that can be built from source (GitLab access) or used online through the biosphere cloud of the IFB (Institut Français de Bioinformatique). The DECOMICS workflow is illustrated in Fig. 1. It includes a guide section, which serves as materials and methods, and two main modules: the deconvolution module and the biological interpretation module (Fig. 1A and B).

### 2.1 Deconvolution module

The deconvolution module (Fig. 1C) is used to load the data, carry out the preprocessing, estimate the number of components, run the deconvolution, and visualize the results. File input (i) requires a .csv file containing the omic data (either gene expression or DNAm) with samples in columns and features in rows. Gene expression can be in the form of raw counts, processed counts, or processed gene expression in the case of microarray-based technologies. We offer basic preprocessing features for gene expression data in step 2. DNAm data should be provided in the form of β-values. Basic preprocessing (ii) of the gene expression data can be achieved within the application, including normalization adapted to RNA-seq data (Read-per-million or *DESeq2*) and transformation (log2 or pseudoLog). Preprocessing also includes the option to select a subset of features. Specifically, one can choose the top 1000 or 5000 gene expressions, or the top 10 000 or 20 000 β-values, based on the highest coefficients of variation. The number of components (iii) to infer (corresponding to the deconvoluted components) has to be estimated by the user. In general, the optimal number of components can be identified through various methods, including Cattell’s rule applied to principal component analysis (PCA) eigenvalues (Cattell 1966), the minimum description length (MDL; Chen *et al.* 2020), bootstrapping techniques (Houseman *et al.* 2016), and cross-validation methods (Lutsik *et al.* 2017). However, in a prior benchmark analysis, we found that different methods yielded comparable results (Decamps *et al.* 2020). For the sake of clarity, we have chosen to present a single method in the application: a guidance plot based on PCA eigenvalues. Then deconvolution (iv) is run by one of the six unsupervised algorithms provided in the application. Depending on the type of omic data provided, a subset of algorithms is available: (i) ICA, NMF, CDSeq, debCAM, and PREDE for gene expression data, and (ii) ICA, NMF, debCAM, and EDec for DNAm data. Finally, deconvolution results (v) can be visualized by (i) a “cell-type” signature heatmap displaying the five top markers of each component, and (ii) a “cell-type” proportion heatmap of each component.

### 2.2 Biological interpretation module

The biological interpretation module (Fig. 1D) performs an enrichment analysis on the components estimated by the chosen deconvolution algorithm and displays the deconvoluted proportion matrix. First, we propose an enrichment analysis (vi) step, to help with the biological interpretation of the components identified by unsupervised approaches. It proposes to perform enrichment analysis [gene set enrichment analysis (GSEA) or over-representation analysis (ORA)] using various biological databases: “CellMatch” (Shao *et al.* 2020), a reference database derived from various resources and other reference ones (GO, GTEx, KEGG, Reactome, Tissue Cell Types, Cancer Cell Types, and Cancer Cell lines). Second, a proportion visualization (vii) section offers the possibility to visualize the full component distribution for a single sample, or the distribution of a single component throughout the total cohort.

## 3 Material and methods

### 3.1 Deconvolution algorithms

Unsupervised deconvolution problem applied to omic bulk data consists in solving an equation of form X=A×T where *T* and *A* are jointly inferred from *X*. This is achieved by estimating the mixture of *K* cell-types, present in different proportions in each sample (cell-type proportion matrix A). Therefore, *X* can be described as a combination of cell-type specific molecular profiles (cell-type specific gene expression matrix *T*). The specifics of each existing unsupervised deconvolution algorithm and the reasons for choosing to include them or not in the DECOMICS application are presented in Supplementary Table S1. In DECOMICS, we provide six different algorithms to run deconvolution: ICA is a blind source separation algorithm that decomposes signal into statistically independent components. In DECOMICS, ICA deconvolution is run using fastICA (R CRAN) and the Deconica (Czerwinska 2018) R packages. By default, 30 significant gene markers are selected to get component scores, using the “weighted.mean” summary metric.

- NMF (Gaujoux and Seoighe 2010): In NMF approach, the molecular profile matrix X is factorized into two matrices A and T, with the property that all three matrices have no negative elements. DECOMICS uses the R CRAN NMF package with method = “snmf/r.” The estimated A matrix is constrained to sum the proportion to 1, and *T* is computed as $T=A^{-1}X$ using the ginv inverse function from MASS R package. Finally, all negative values for *T* are set to 0.
- CDSeq (Kang *et al.* 2021) aims at simultaneously estimating A and T matrices using a probabilistic model based on latent Dirichlet allocation (LDA). DECOMICS uses the R implementation of the CDseq method CDSeqR with the following parameters: beta = 0.5, alpha = 5, mcmc_iterations = 300. The reduction factor is computed to avoid expression values $>10^{5}$; block numbers and gene block size are computed such that a block does not exceed $10^{3}$ genes.
- debCAM (Chen *et al.* 2020) stands for deconvolution by Convex Analysis of Mixtures. This method uses a geometric approach to identify a solution to the NMF problem in the simplex space. Thus, the proposed solution for A is always a proportion matrix. In DECOMICS, the function CAM is called from the debCAM R packages using the following empirical parameters: cluster.num is computed to be five times greater than the number of expected components, and dim.rdc set to divide the number of input genes by a tenth.
- PREDE (Qin *et al.* 2020) is a method that offers the possibility to conduct partial reference-based deconvolution method solved via an iterative Quadratic Programming procedure. In DECOMICS, PREDE function is used from PREDE R package, with the following parameters: W1 = NULL (which corresponds to a complete deconvolution approach), type = “GE,” iters = 100 and rssDiffStrop = 1e−5.
- EDec-step1 (Onuchic *et al.* 2016) estimates both average component methylation profiles and component proportions using an iterative constrained matrix factorization algorithm. This algorithm identifies cell type-specific methylation profiles and constituent cell type proportions by minimizing the Euclidean distance between the reconstituted and original mixed methylation matrices. In DECOMICS, we use the EDec::run_edec_stage_1 function with the parameters max_its = 2000 and rss_diff_stop = 1e−10.

### 3.2 Gene set enrichment analysis

In order to biologically characterize each of the unsupervised components identified, biological enrichment analyses are performed. For each component, the first step consists in ranking the genes according to their coordinates on the component, in order to identify the most contributing genes of the component. For methylation data, CpG coordinates are aggregated at the gene level, taking the maximum value observed for CpGs of the same gene. This approach is a way of considering a gene as strongly contributing to the component if it has at least one strongly contributing CpG. In a second step, an enrichment analysis is performed either based on GSEA (Mootha *et al.* 2003) or ORA (Goeman and Bühlmann 2007). If the coordinates contain sufficient nonduplicate values (threshold set at 30% by default) to enable reliable ordering of the values, a GSEA analysis is performed using the fgsea R package (Korotkevich *et al.* 2021); otherwise, an ORA analysis is performed, taking as gene selection the top 20% of the component’s most contributing genes and as gene universe all the genes available in the user’s dataset. Various biological databases can be queried. DECOMICS includes the CellMatch database restricted to the human species and cell types with at least three marker genes per cell type. After filtering, it provides marker genes for 120 different cell types across 103 normal tissues and 26 tumoral tissues. DECOMICS also includes the latest versions of the following biological databases provided on the Enrichr (Chen *et al.* 2013) tool website:

- Cancer Cell Line Encyclopedia (967 terms)
- CellMarker Augmented 2021 (1097 terms)
- GO Biological Process 2023 (5407 terms)
- GO Cellular Component 2023 (474 terms)
- GO Molecular Function 2023 (1147 terms)
- GTEx Tissues V8 2023 (511 terms)
- KEGG 2021 Human (320 terms)
- MSigDB Oncogenic Signatures (189 terms)
- NCI 60 Cancer Cell Lines (93 terms)
- Reactome 2022 (1818 terms)

Prior to the enrichment analysis, components obtained from the ICA-based method are reoriented, following the approach proposed in the deconica R package, which is based on the hypothesis that the highest absolute values of a component's weight should be positive.

## 4 Availability

Installation instructions can be found on the DECOMICS gitlab webpage: https://gitlab.in2p3.fr/Magali.Richard/decomics. There are three installation options: (i) full local installation, (ii) running locally a virtual machine (VM), or (iii) using the Biosphere-IFB cloud. Local installation requires several packages to be loaded. To help users, we propose a conda recipe on the DECOMICS gitlab webpage. To offer the possibility to run DECOMICS on a local VM, we built a docker container. The user simply needs to install docker on their machine and launch the provided image. Finally, DECOMICS is deployed on the Biosphere portal (searchable through the RAINBio catalog). To use the clouds of IFB-Biosphere, users need to create an account and get membership of an active group (more information can be found here: https://ifb-elixirfr.github.io/biosphere/signin). Then users can deploy and connect to VM using the web interface (tutorial here: https://ifb-elixirfr.github.io/biosphere/vm_connect).

## 5 Application and results

**Figure 2.**
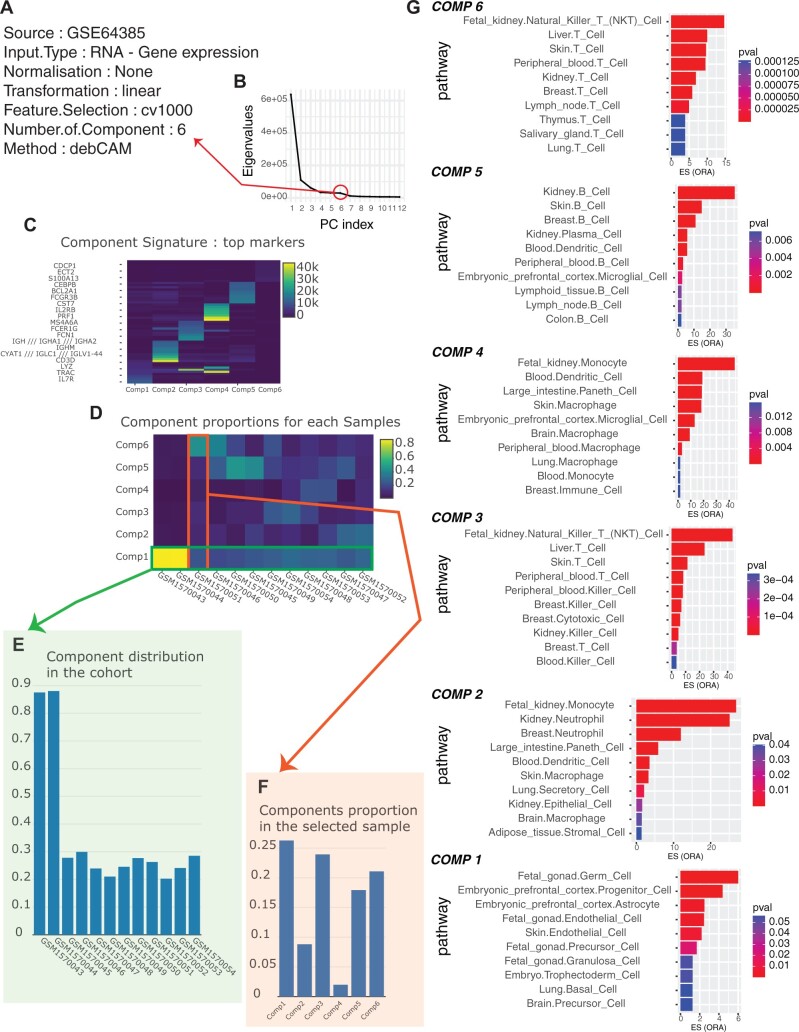
Illustration of DECOMICS-based deconvolution of gene expression data. (A) Parameters used in the DECOMICS application are described. (B) Scree plot illustrating the selection process for the number of components to be deconvolved (module 1, step 3: number of cell types). (C, D) Deconvolution results are presented: (C) component signatures are plotted and (D) a heatmap showing component proportions (module 1, step 5: results). (E, F) Visualization of component distribution: (E) distribution of a specific component across the cohort and (F) distribution of all components within a given sample (module 2, step 7: proportion visualization). (G) Enrichment plot displaying the enrichment analysis for each component (module 2, step 6: enrichment analysis), ORA indicates if an overrepresentation analysis has been performed, and GSEA indicates if a gene set enrichment analysis has been performed for a given sample. *P*val corresponds to the adjusted *P*-values of the enrichment score (ES) after correction for multiple testing.

We have provided two use cases to illustrate the DECOMICS pipeline. The first use case is based on gene expression data (GSE64385; Becht *et al.* 2016). Dataset is available for download in .csv format from the DECOMICS application. This dataset consists of a mixture of six cell types: HTC116, neutrophils, natural killer cells, monocytes, B cells, and T cells. As shown in Fig. 2, the unsupervised components identified by DECOMICS are highly consistent with the constituent cell types. In this example, the deconvolution algorithm employed is debCAM and the functional enrichment analyses were performed using the CellMatch database. The second use case utilizes DNAm profiles from reconstituted mixtures of six purified immune cells derived from human blood samples (GSE77797; Koestler *et al.* 2016). This example further showcases the efficacy of the DECOMICS pipeline for DNAm unsupervised deconvolution and biological interpretation of the unsupervised components. Detailed information on this use case is provided in Supplementary Fig. S1, and the corresponding .csv dataset is available for download from the DECOMICS application. In the DECOMICS pipeline, the input data consist of simple count tables in .csv format, which can be uploaded directly to the application. Example input files demonstrating the input format are available for download from the application. The preprocessed data (.csv), deconvolution results (.rds), top 100 contributing genes of each component (.csv), estimated typical gene expressions for components (.csv), and proportion estimates (.csv) can also be downloaded from the application. Additionally, enrichment analyses can be downloaded in the form of a .csv table containing the enrichment scores and *P*-values for the queried database.

## 6 Discussion

Despite their advantages over supervised methods, the use of unsupervised deconvolution methods is not trivial, as it requires a priori knowledge of the number of cell populations to be considered, as well as a biological interpretation of the estimated components. Here we provide a user-friendly interactive web-application to perform unsupervised deconvolution by assisting the user in the choice of the number of components and biological interpretation of the results. Significant efforts have been made by colleagues to integrate unsupervised deconvolution into analysis pipelines, including determining the number of components to infer and interpret the biological data (Li and Wu 2019, Scherer *et al.* 2020). However, executing these pipelines in their entirety necessitates the installation of R packages and the use of command line interfaces, which our web application avoids. Additionally, these pipelines offer a limited selection of deconvolution methods, restricted to those developed by the authors of the integrative pipeline. In this work, we propose an unbiased approach that incorporates several deconvolution methods. DECOMICS stands out for its ease of use, speed, and comprehensive documentation. It is designed to be accessible to users without expertise in R or biostatistics. With its integrated biological interpretation feature, users can seamlessly perform both deconvolution and interpretation within the same platform, eliminating the need for external tools. Moreover, DECOMICS is highly adaptable, allowing for easy updates and redeployment as new reference-free methods or enrichment analysis databases become available. This flexibility ensures that DECOMICS remains at the cutting edge, capable of incorporating the latest technical and methodological advancements.

## Supplementary Material

vbae136_Supplementary_Data

## Data Availability

The data underlying this article are available in the GEO public repository under the accession numbers: GSE64385 and GSE77797 (https://www.ncbi.nlm.nih.gov/geo/).
