# Supplementary material for: DECOMICS, a shiny application for unsupervised cell type deconvolution and biological interpretation of bulk omic data
Source: Bioinform Adv. 2024 Sep 20;4(1):vbae136. doi: 10.1093/bioadv/vbae136 (PMC11479579; doi:10.1093/bioadv/vbae136)
Supplement: vbae136_Supplementary_Data [file vbae136_supplementary_data.pdf]

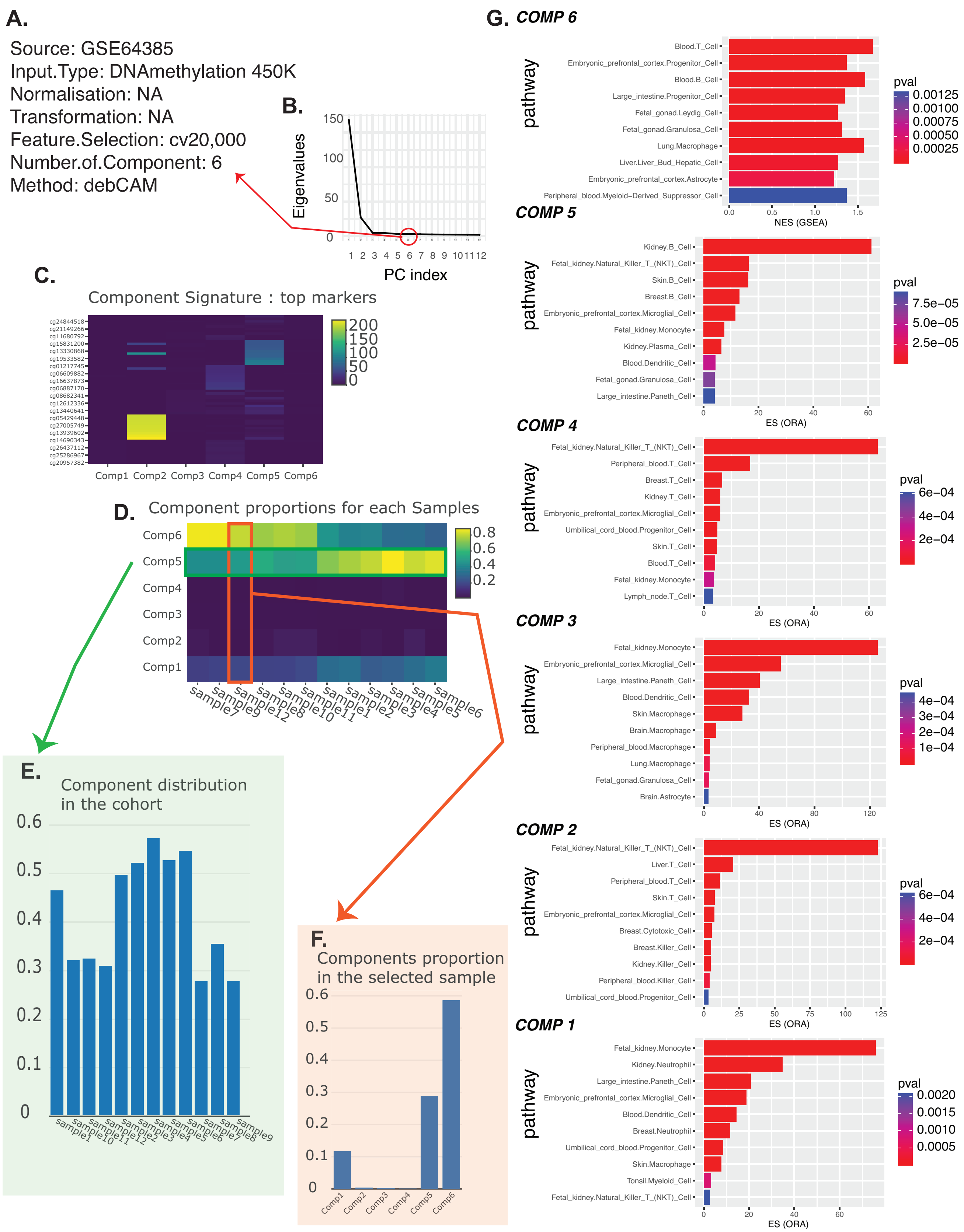

Supplementary Figure 1. Illustration of DECOMICS based deconvolution of DNA methylation data. A. Parameters used in the DECOMICS application are described. B. Scree plot illustrating the selection process for the number of components to be deconvolved (module 1, step 3: Number of cell types). C-D. Deconvolution results are presented: (C) Component signatures are plotted, and (D) a heatmap showing component proportions (module 1, step 5: Results). E-F. Visualization of component distribution: (E) Distribution of a specific component across the cohort, and (F) distribution of all components within a given sample (module 2, step 7: Proportion visualization). G. Enrichment plot displaying the enrichment analysis for each component (module 2, step 6: Enrichment analysis) ORA indicates if an Overrepresentation Analysis has been performed, GSEA indicates if a Gene Set Enrichment Analysis has been performed for a given sample. Pval corresponds to the adjusted p-values of the Enrichment Score (ES) after correction for multiple testing.

Table 1. Descriptive table of existing unsupervised deconvolution algorithm. This table provides a brief overview of each algorithm, highlighting their methodologies and key features. The citation count was obtained in July 2024.

**Methods**	**Approach**	**Omic type**				**Nb of citations**	**OS**	**Package accessibility**	**Imports**	**Depends**	**URL**	**Included in DECOMICS**
ICA	ICA	both	R	2023	DOI	NA	NA	cran		R (≥ 4.0.0)	URL	Yes
NMF	NMF	both	R	2010	DOI	640	NA	cran	graphics, stats, stringr (≥ 1.0.0), digest, grid, grDevices, gridBase, colorspace, RColorBrewer, foreach, doParallel, ggplot2, reshape2, Biobase, codetools, BiocManager	R (≥ 3.0.0), methods, utils, registry, rngtools (≥ 1.2.3), cluster	URL	Yes
MeDeCom	NMF + constrains	DNAm	R	2017	DOI	64	Only binary release accessible for MacOS, docker image for windows	github	RcppEigen (≥ 0.3)	R (≥ 3.2.0), Rcpp, pracma, gtools, gplots, parallel, RUnit, RnBeads	URL	No (problem of OS compatibilities)
debCAM	Convex analysis	both	R	2020	DOI	9	NA	bioconductor	methods, rJava, BiocParallel, stats, Biobase, SummarizedExperiment, corpcor, geometry, NMF, nnls, DMwR2, pcaPP, apcluster, graphics	R (≥ 3.5)	URL	Yes
RefFreeEWAS/ RefFreeCellMix	NMF	DNAm	R	2016	DOI	74	NA	removed from cran in 2021	NA	NA	NA	No, no longer accessible on CRAN (but similar to EDec_step1)
EDec_step1	NMF, equivalent to RefFreeEWAS	DNAm	R	2016	DOI	51	NA	github	quadprog, gtools, stats, clue, utils	R (≥ 3.1.2)	URL	Yes
PREDE	NMF	RNA	R	2020	DOI	5	NA	github		R (≥ 3.5.0), matrixStats, stats, utils, quadprog, gtools	URL	Yes (only reference-free option)
CDSeqR	Probabilistic model	RNA	R	2021	DOI	3	NA	github	Rcpp (≥ 1.0.3), MASS, foreach, doParallel, dirmult, RcppThread, iterators, parallel, grDevices, Seurat, ggplot2, magrittr, dplyr, rlang, Matrix, matrixStats, ggpubr, pheatmap, harmony	R (≥ 3.6.0)	URL	Yes
Tsisal	SISAL (geometric algorithm)	DNAm	R	2021	DOI	4	NA	bioconductor	stats, methods, SummarizedExperiment, corpcor, doParallel, parallel, ggplot2, tidyr, Ggally	R (≥ 3.6), EpiDISH, limma, nnls, quadprog	URL	No, because the method doesn't generate the component profiles in output, rather, it only includes selected cell type-specific markers.
PRMeth	NMF	DNAm	R	2022	DOI	1	NA	not a package			URL	No (no R package avalaible)
CAM3	Convex analysis	both	R	2024	DOI	0	NA	github	methods, debCAM, stats, Biobase, SummarizedExperiment, corpcor, geometry, NMF, nnls, graphics, geometry	R (≥ 3.6)	URL	in beta channel
